# Emerging Prognostic Risk Factors in Acute Coronary Syndromes: Beyond Traditional Risk Assessment

**DOI:** 10.3390/jcm15187312

**Published:** 2026-09-20

**Authors:** Lorenzo Cangiano, Giancarlo Marenzi, Gianluca Pontone, Nicola Cosentino

**Affiliations:** 1 Centro Cardiologico Monzino, IRCCS, 20138 Milan, Italy; lorenzo.cangiano@cardiologicomonzino.it (L.C.); giancarlo.marenzi@ccfm.it (G.M.); gianluca.pontone@ccfm.it (G.P.); 2 Department of Perioperative Cardiology and Cardiovascular Imaging, Centro Cardiologico Monzino IRCCS, 20138 Milan, Italy; 3 Department of Biomedical, Surgical and Dental Sciences, University of Milan, 20138 Milan, Italy

**Keywords:** acute coronary syndrome, risk factors, risk assessment, prognosis

## Abstract

Acute coronary syndromes (ACS) remain a major cause of cardiovascular mortality and long-term morbidity worldwide despite substantial advances in revascularization techniques, antithrombotic therapies, and lipid-lowering strategies. Although contemporary management has significantly improved survival, a considerable residual risk of recurrent ischemic events, adverse ventricular remodelling, heart failure, and cardiovascular death persists following ACS. Consequently, accurate risk stratification has become increasingly important to guide individualized secondary prevention and optimize long-term outcomes. Traditional risk assessment after ACS relies largely on clinical characteristics, conventional cardiovascular risk factors, and established risk scores such as GRACE and TIMI. However, growing evidence indicates that several emerging biomarkers and pathophysiological pathways provide additional prognostic information beyond conventional models. These markers reflect distinct biological processes including residual inflammatory activity, myocardial injury and remodelling, renal dysfunction, metabolic impairment, and visceral adiposity. This review summarizes current evidence regarding both established and emerging prognostic markers after ACS across six major pathophysiological domains: lipid abnormalities and traditional cardiovascular risk factors; systemic inflammation and residual inflammatory risk; myocardial injury and adverse ventricular remodelling; cardiorenal dysfunction; visceral adiposity; and anti-inflammatory therapeutic strategies. For each domain, we discuss underlying biological mechanisms, key findings from major clinical trials and registries, and the potential incremental value of biomarkers in contemporary risk stratification. Collectively, these markers support a multidimensional approach to post-ACS risk assessment and may contribute to more personalized therapeutic strategies aimed at reducing residual cardiovascular risk.

## 1. Introduction

Despite remarkable therapeutic advances over recent decades, cardiovascular disease (CVD) remains the leading cause of mortality and disability worldwide. According to the most recent Global Burden of Disease analysis, CVD accounted for nearly 20 million deaths globally in 2022 and continues to represent the principal contributor to years of life lost worldwide [1]. Although age-standardized cardiovascular mortality has progressively declined, the absolute burden of disease continues to increase because of population ageing, increasing prevalence of cardiometabolic risk factors, and improved survival following acute cardiovascular events [1,2]. Furthermore, substantial geographic and socioeconomic disparities persist, with disproportionately higher cardiovascular mortality observed in low- and middle-income countries [2].

Acute coronary syndromes (ACS) frequently represent the first clinical manifestation of coronary artery disease and remain among the most important contributors to cardiovascular mortality and morbidity worldwide. Beyond the acute ischemic event, ACS is associated with a broad spectrum of long-term complications including recurrent myocardial infarction, heart failure, arrhythmias, repeated hospitalizations, impaired quality of life, and increased healthcare utilization. Despite widespread implementation of early invasive management strategies, percutaneous coronary intervention (PCI), high-intensity statin therapy, PCSK9 inhibition, and more potent antithrombotic regimens, a substantial proportion of patients remain exposed to significant residual cardiovascular risk. The persistence of recurrent events despite guideline-directed therapy highlights the complex and multifactorial nature of atherosclerotic disease. Residual risk following ACS extends beyond cholesterol-mediated mechanisms and includes persistent inflammatory activation, myocardial injury and remodelling, renal dysfunction, metabolic abnormalities, neurohormonal activation, and adverse body fat distribution. Consequently, traditional risk factors alone may not fully capture the biological heterogeneity that characterizes patients after ACS.

Accurate prognostic stratification has therefore become a central component of contemporary post-ACS management. Current European and American guidelines [3,4] increasingly advocate individualized treatment approaches, particularly regarding the duration and intensity of dual antiplatelet therapy, lipid-lowering interventions, and implementation of secondary prevention measures. Early identification of patients at highest risk may facilitate more intensive therapeutic strategies, closer surveillance, and earlier implementation of interventions aimed at preventing recurrent ischemic events and heart failure. The clinical relevance of improved risk stratification is further supported by evidence suggesting that the absolute benefit of intensified therapies is greatest among patients with the highest baseline risk. Biomarker-based analyses have demonstrated that multi-marker approaches can identify patient subsets deriving greater benefit from potent antiplatelet therapies [5]. Paradoxically, however, patients with the highest ischemic risk are frequently less likely to receive intensive treatment strategies, a phenomenon commonly described as the treatment-risk paradox [6]. Advanced age, frailty, renal dysfunction, concerns regarding bleeding risk, and therapeutic inertia may all contribute to this discrepancy. Traditional cardiovascular risk factors remain fundamental determinants of prognosis after ACS (Table 1) and continue to underpin established risk prediction models such as Global Registry of Acute Coronary Events (GRACE) and The Thrombolysis in Myocardial Infarction (TIMI). Nevertheless, increasing evidence suggests that several emerging biomarkers reflecting inflammation, myocardial stress, renal dysfunction, fibrosis, and metabolic impairment may provide incremental prognostic information beyond conventional risk assessment tools. Understanding the prognostic significance of these markers may facilitate a more comprehensive and biologically informed approach to risk stratification.

In this review, we critically examine both established and emerging prognostic markers in ACS, focusing on their pathophysiological basis, clinical evidence, and potential role in multidimensional risk stratification. Particular emphasis is placed on biomarkers and mechanisms that may improve identification of residual cardiovascular risk and support the development of personalized secondary prevention strategies. Throughout the review, we have endeavoured to prioritize evidence from randomized controlled trials and large prospective cohort studies and to distinguish it from retrospective analyses and exploratory biomarker data; where the supporting evidence is predominantly observational or mechanistic in nature, this is acknowledged either within the relevant section or in the Limitations. In particular, we highlight evidence regarding incremental prognostic value beyond established risk factors and risk scores, when present, as well as the potential integration of selected biomarkers into clinical risk stratification and decision-making. For most emerging biomarkers, however, current evidence remains primarily focused on prognostic associations, and their incremental value, clinical utility, and applicability in routine practice remain incompletely established. By bringing together the available evidence across different biological domains, this review may help identify promising candidates for future studies aimed at determining which emerging biomarkers provide meaningful incremental prognostic value and, ultimately, clinically actionable information beyond established risk assessment.

## 2. Dyslipidaemia and Traditional Cardiovascular Risk Factors

### 2.1. Low-Density Lipoprotein Cholesterol

Low-density lipoprotein cholesterol (LDL-C) remains the cornerstone lipid biomarker in cardiovascular prevention and one of the most important determinants of atherosclerotic cardiovascular disease. The causal relationship between LDL particles and atherosclerosis is supported by extensive experimental, genetic, epidemiological, and interventional evidence. Following retention within the arterial intima, LDL particles undergo oxidative and enzymatic modification, triggering endothelial dysfunction, activation of innate and adaptive immune responses, recruitment of inflammatory cells, and foam-cell formation. These processes drive plaque growth, promote plaque instability, and ultimately predispose to thrombotic complications resulting in ACS [7]. Consequently, LDL-C reduction has become a central therapeutic target in both primary and secondary prevention [3,4]. Multiple randomized clinical trials have demonstrated a strong association between LDL-C lowering and reductions in recurrent ischemic events, supporting the concept that cardiovascular benefit is proportional to the absolute magnitude of LDL-C reduction [8,9]. The introduction of PCSK9 inhibitors further reinforced the “lower is better” paradigm. In both the Further Cardiovascular Outcomes Research With PCSK9 Inhibition in Subjects With Elevated Risk (FOURIER) and ODYSSEY OUTCOMES trials, achievement of very low LDL-C concentrations through PCSK9 inhibition was associated with additional reductions in cardiovascular events beyond those obtained with intensive statin therapy alone [10,11]. Nevertheless, substantial residual cardiovascular risk persists despite aggressive lipid-lowering treatment. This observation highlights that LDL-C alone does not fully capture the complexity of post-ACS risk [12]. Patients with similar LDL-C levels may exhibit markedly different clinical trajectories due to the influence of other biological pathways including inflammation, myocardial injury, renal dysfunction, and metabolic abnormalities. Furthermore, the prognostic impact of LDL-C appears heterogeneous across patient populations. While individuals presenting with higher LDL-C concentrations derive greater absolute benefit from intensive lipid-lowering strategies [11], several observational studies have paradoxically reported worse outcomes among patients presenting with lower LDL-C levels during ACS [13]. This phenomenon, often referred to as the “cholesterol paradox,” likely reflects the coexistence of frailty, malnutrition, chronic inflammation, or advanced comorbidity rather than a protective effect of elevated cholesterol itself.

Taken together, these observations suggest that LDL-C remains an indispensable therapeutic target but is insufficient as a standalone prognostic marker after ACS. Comprehensive risk assessment therefore requires integration of lipid-related risk with additional biological pathways contributing to residual cardiovascular risk.

### 2.2. Lipoprotein(a)

Among lipid-related risk factors, lipoprotein(a) [Lp(a)] has emerged as one of the most promising biomarkers of residual cardiovascular risk. Structurally, Lp(a) consists of an LDL-like particle containing apolipoprotein B-100 covalently bound to apolipoprotein(a), a highly polymorphic glycoprotein encoded by the LPA gene. Unlike LDL-C, circulating Lp(a) concentrations are predominantly genetically determined and remain relatively stable throughout life. Lp(a) exerts multiple potentially harmful effects that extend beyond cholesterol transport. Experimental and clinical evidence supports proatherogenic, pro-inflammatory, pro-oxidative, and prothrombotic properties. Through promotion of endothelial dysfunction, enhanced oxidative modification of lipoproteins, and interference with fibrinolytic pathways, elevated Lp(a) may contribute to both plaque progression and thrombosis. While elevated Lp(a) is well established as a risk factor for incident cardiovascular disease [14], increasing attention has focused on its prognostic role after ACS. A recent meta-analysis demonstrated that elevated Lp(a) concentrations were independently associated with increased risks of major adverse cardiovascular events and all-cause mortality among ACS patients [15]. These findings suggest that Lp(a) may identify a subgroup of patients characterized by persistent residual risk despite contemporary secondary prevention strategies.

The therapeutic implications of Lp(a) remain an area of intense investigation. Conventional lipid-lowering therapies have limited effects on Lp(a) concentrations. Statins generally do not reduce Lp(a) levels and may occasionally increase them modestly. In contrast, PCSK9 inhibitors consistently reduce Lp(a) concentrations in addition to lowering LDL-C. Secondary analyses from both FOURIER and ODYSSEY OUTCOMES suggest that part of the cardiovascular benefit observed with PCSK9 inhibition may be attributable to reductions in Lp(a), particularly among patients with elevated baseline concentrations [16,17]. However, the extent to which Lp(a) reduction independently contributes to improved outcomes remains uncertain. Ongoing dedicated Lp(a)-lowering trials employing novel RNA-targeted therapies will be critical to establish whether Lp(a) represents not only a prognostic biomarker of residual risk but also a genuinely modifiable therapeutic target; if confirmed, this would represent a paradigm shift in secondary prevention after ACS, extending lipid-lowering strategies beyond LDL-C to encompass a genetically determined and previously untreatable driver of residual cardiovascular risk.

### 2.3. Cigarette Smoking

Cigarette smoking remains one of the most important modifiable cardiovascular risk factors and contributes to adverse outcomes through multiple biological mechanisms. Exposure to tobacco smoke promotes endothelial dysfunction, oxidative stress, vascular inflammation, platelet activation, impaired fibrinolysis, and accelerated atherosclerotic progression. By reducing nitric oxide bioavailability and increasing production of reactive oxygen species, smoking creates a vascular environment characterized by enhanced thrombogenicity and plaque vulnerability [18,19]. Historically, several studies reported apparently improved short-term outcomes among smokers presenting with acute myocardial infarction (AMI), giving rise to the concept of the “smoker’s paradox” [20,21]. Subsequent analyses demonstrated that this observation was largely explained by differences in baseline characteristics, particularly younger age and lower comorbidity burden among smokers. After appropriate adjustment, the apparent survival advantage disappears, leading to the concept of the “smoker’s pseudoparadox” [22,23]. Despite resolution of the paradox, the relationship between smoking and long-term prognosis after ACS remains complex. Contemporary studies have shown that smoking is associated with increased risk of recurrent myocardial infarction and adverse cardiovascular events [22]. More importantly, advanced imaging studies have demonstrated that smokers presenting with STEMI exhibit enhanced inflammatory activation, greater infarct-zone haemorrhage, and worse long-term cardiac outcomes [23]. These findings suggest that smoking may exert particularly deleterious effects on myocardial injury and post-infarction remodelling, extending its impact beyond atherosclerotic disease alone. Accordingly, smoking cessation remains one of the most effective interventions for reducing recurrent cardiovascular risk after ACS and should represent a central component of secondary prevention programs.

### 2.4. Hypertension

Hypertension is a major determinant of cardiovascular risk throughout the continuum of coronary artery disease and remains highly prevalent among patients presenting with ACS. Persistent elevation of blood pressure promotes endothelial dysfunction, arterial stiffness, vascular remodelling, chronic inflammatory activation, and microvascular dysfunction, thereby accelerating atherosclerotic progression and increasing susceptibility to ischemic events [24]. Beyond its role in the development of coronary disease, hypertension also appears to influence prognosis following ACS. Contemporary evidence indicates that antecedent hypertension is associated with increased long-term mortality and higher rates of heart failure after myocardial infarction [25,26,27]. Although improvements in antihypertensive therapy and secondary prevention have attenuated this association over time, hypertension continues to identify patients at elevated residual risk [27]. Interestingly, the adverse prognostic impact of hypertension does not appear to be explained by larger infarct size or more extensive acute reperfusion injury. Cardiac magnetic resonance studies have demonstrated similar infarct size and comparable degrees of microvascular obstruction in hypertensive and normotensive patients [26,28,29]. However, hypertensive individuals exhibit greater vascular degradation and increased susceptibility to haemorrhagic transformation within the infarct zone. These observations suggest that chronic vascular damage preceding ACS may contribute to adverse ventricular remodelling and worse long-term outcomes independently of the magnitude of the acute ischemic injury [26].

### 2.5. Diabetes Mellitus

Among traditional cardiovascular risk factors, diabetes mellitus exerts one of the most profound influences on prognosis after ACS. Chronic hyperglycaemia induces a broad spectrum of pathological alterations including endothelial dysfunction, oxidative stress, inflammation, platelet activation, impaired fibrinolysis, and accelerated atherosclerosis. In addition, diabetes promotes diffuse coronary artery disease, microvascular dysfunction, and adverse myocardial remodelling, all of which contribute to poorer outcomes after an ischemic event [30]. The prognostic impact of diabetes after ACS has been consistently demonstrated across multiple studies. In a pooled analysis of more than 62,000 patients enrolled in eleven TIMI trials, diabetes was independently associated with increased 30-day mortality across the entire spectrum of ACS presentations. Notably, one-year mortality among diabetic patients presenting with UA/NSTEMI approached that observed in non-diabetic patients presenting with STEMI, emphasizing the substantial risk associated with the diabetic phenotype irrespective of ACS subtype [31]. Importantly, diabetes-related risk appears to be duration-dependent. In a contemporary cohort exceeding 138,000 patients with AMI, both in-hospital and one-year mortality increased progressively with longer diabetes duration, reaching significantly higher levels among patients with disease duration exceeding ten years [32]. These findings suggest that cumulative exposure to metabolic dysfunction contributes importantly to adverse cardiovascular outcomes. Despite major advances in revascularization and antithrombotic therapy, the excess risk associated with diabetes persists. This observation indicates that mechanisms extending beyond acute ischemia, including inflammation, metabolic impairment, microvascular dysfunction, and myocardial remodelling, continue to influence prognosis [33]. Furthermore, the traditional binary classification of diabetic versus non-diabetic status likely oversimplifies the biological heterogeneity present within this population. Future risk stratification strategies may benefit from incorporating measures of glycaemic control, insulin resistance, and diabetes-related organ damage to more accurately capture metabolic risk after ACS.

## 3. Inflammation and Residual Inflammatory Risk

Inflammation is now recognized as a fundamental driver of atherosclerosis and ACS. Rather than representing a passive lipid-storage disorder, atherosclerosis is increasingly understood as a chronic inflammatory disease characterized by complex interactions between immune cells, vascular cells, lipoproteins, and circulating inflammatory mediators. Throughout plaque development, activated macrophages, monocytes, T lymphocytes, endothelial cells, and smooth muscle cells participate in a dynamic inflammatory process that promotes plaque progression, destabilization, and eventual thrombotic complications. The occurrence of ACS itself triggers a profound inflammatory response extending beyond the culprit lesion. Following myocardial ischemia and necrosis, inflammatory pathways become activated both locally and systemically, contributing not only to tissue repair but also to adverse ventricular remodelling and recurrent cardiovascular events. Increasing evidence suggests that persistent inflammatory activation after ACS may represent a major component of residual cardiovascular risk, independent of lipid levels and traditional risk factors (Table 2) [34].

### 3.1. Residual Inflammatory Risk and High-Sensitivity C-Reactive Protein

The concept of residual inflammatory risk emerged from the observation that many patients continue to experience recurrent cardiovascular events despite achieving guideline-recommended LDL-C targets. This concept has been particularly strengthened by analyses demonstrating that inflammatory biomarkers remain independently associated with adverse outcomes even among patients receiving intensive lipid-lowering therapy [35]. Among available inflammatory biomarkers, high-sensitivity C-reactive protein (hsCRP) remains the most extensively studied and clinically validated. Synthesized by the liver in response to interleukin-6 stimulation, hsCRP serves as an integrated marker of systemic inflammatory activation. Although not directly involved in all aspects of atherogenesis, hsCRP reflects activation of inflammatory pathways strongly associated with cardiovascular risk. Compelling evidence supporting residual inflammatory risk emerged from analyses of the FOURIER trial [35]. Despite intensive LDL-C reduction with evolocumab, both achieved LDL-C and baseline hsCRP independently predicted future cardiovascular events. Patients with hsCRP concentrations greater than 3 mg/L experienced nearly 50% higher three-year rates of the primary cardiovascular endpoint compared with those with hsCRP levels below 1 mg/L, irrespective of lipid-lowering efficacy. These findings provided strong evidence that inflammation contributes independently to residual cardiovascular risk. Observational studies have consistently confirmed the prognostic value of hsCRP following ACS. In a large contemporary cohort, increasing hsCRP concentrations were associated with progressively higher long-term mortality independently of troponin levels, demonstrating that inflammatory activity provides prognostic information distinct from myocardial necrosis [36]. Furthermore, persistent hsCRP elevation after ACS has been linked to increased risks of recurrent ischemic events, heart failure, restenosis, and all-cause mortality [37,38].

It should be noted, however, that hsCRP concentrations measured during the acute phase of ACS require cautious interpretation. Although the relatively delayed kinetics of hsCRP may limit the influence of myocardial necrosis in patients presenting early after symptom onset, delayed presentation may result in hsCRP levels that predominantly reflect infarct size, tissue necrosis, procedural injury, or intercurrent inflammatory conditions rather than residual inflammatory risk. Conversely, studies assessing hsCRP beyond the acute phase have consistently demonstrated its ability to capture persistent low-grade inflammation associated with adverse cardiovascular outcomes. Therefore, hsCRP should be regarded as a biomarker reflecting both the severity of the acute event and residual inflammatory risk, with its prognostic interpretation depending on the timing of measurement.

Collectively, these findings suggest that inflammation should not be viewed merely as a marker of disease burden but rather as an active contributor to adverse cardiovascular outcomes. Persistent inflammatory activation may therefore identify a biologically distinct population characterized by heightened residual risk despite optimal conventional therapy.

### 3.2. Interleukin-6 and Upstream Inflammatory Signalling

While hsCRP represents a downstream marker of inflammation, increasing attention has focused on upstream mediators that may play more direct mechanistic roles in atherosclerotic disease progression. Among these, interleukin-6 (IL-6) has emerged as one of the most important inflammatory biomarkers in cardiovascular medicine. IL-6 is produced by activated leukocytes, endothelial cells, vascular smooth muscle cells, and other tissues in response to vascular injury and oxidative stress. Through stimulation of hepatic acute-phase protein synthesis, endothelial activation, leukocyte recruitment, and coagulation pathways, IL-6 occupies a central position within the inflammatory cascade linking atherosclerosis to clinical events. Numerous studies have demonstrated a strong association between elevated IL-6 levels and adverse outcomes following ACS. Higher circulating concentrations have consistently been associated with increased risks of cardiovascular mortality, recurrent myocardial infarction, and heart failure. These observations have subsequently been confirmed in several meta-analyses, reinforcing the prognostic significance of IL-6 across different ACS populations [39,40,41,42]. The biological relevance of IL-6 is further supported by therapeutic evidence. The Canakinumab Anti-inflammatory Thrombosis Outcomes Study (CANTOS) provided the first definitive proof that targeted inhibition of inflammation can reduce cardiovascular events independently of lipid lowering. By inhibiting interleukin-1β, a cytokine located upstream of IL-6 signalling, canakinumab significantly reduced recurrent cardiovascular events among patients with prior myocardial infarction and persistent residual inflammatory risk. The benefit occurred without significant effects on LDL-C levels, establishing inflammation as an independent therapeutic target in atherosclerotic disease [43]. These findings strongly support the pathogenic importance of the IL-1β/IL-6 inflammatory axis and suggest that inflammatory biomarkers may eventually serve not only as prognostic markers but also as tools for identifying patients most likely to benefit from targeted anti-inflammatory interventions. Nonetheless, like hsCRP, IL-6 concentrations in the acute phase of ACS are confounded by infarct-related tissue injury, haemodynamic stress, and intercurrent inflammatory stimuli, limiting its specificity as a marker of chronic residual inflammatory risk when measured at admission alone.

### 3.3. Leukocyte-Derived Inflammatory Indices

The increasing availability of routine hematological parameters has stimulated interest in simple leukocyte-derived indices as markers of systemic inflammatory activation. These indices integrate information from different components of the immune response and provide inexpensive, readily available tools for risk stratification. Among them, the neutrophil-to-lymphocyte ratio (NLR) has attracted particular attention. Elevated NLR reflects both enhanced innate inflammatory activation, represented by neutrophilia, and impaired adaptive immune regulation, reflected by relative lymphopenia. This combination appears particularly relevant in ACS, where both excessive inflammation and dysregulated immune responses contribute to adverse outcomes.

Multiple studies have demonstrated that elevated NLR is associated with increased short- and long-term mortality after STEMI and ACS. Higher NLR values have also been linked to reduced left ventricular ejection fraction, adverse ventricular remodelling, greater coronary disease complexity, and increased risk of left ventricular apical thrombus formation. Meta-analytic evidence further supports its independent prognostic value across diverse patient populations [44,45,46,47,48]. Additional leukocyte-derived indices have also shown promise. The platelet-to-lymphocyte ratio (PLR), monocyte-to-lymphocyte ratio (MLR), systemic immune-inflammation index (SII), and systemic inflammation response index (SIRI) have each demonstrated independent associations with adverse cardiovascular outcomes after ACS. Particularly noteworthy is emerging evidence suggesting that incorporation of SIRI into traditional risk models such as the GRACE score may further improve prediction of major adverse cardiovascular events [49]. The attractiveness of these indices lies not only in their prognostic performance but also in their universal availability, low cost, and ease of implementation in routine clinical practice.

It is important to note that the prognostic significance of leukocyte-derived indices may depend on the timing of blood sampling after ACS. Values obtained during the early phase may primarily reflect the acute inflammatory response to myocardial injury, whereas persistent or evolving abnormalities on serial assessment may provide different information regarding residual inflammatory risk. Emerging data suggest that the timing of measurement, and potentially changes in leukocyte-derived indices over time, may influence their prognostic performance. However, the optimal timing and clinical relevance of serial measurements remain incompletely established [50].

Beyond these temporal considerations, leukocyte-derived indices are subject to important confounders, including acute infection, physiological stress, corticosteroid treatment, haematological disorders, and malignancy. Their optimal cutoffs and measurement time points vary substantially among studies, limiting direct comparisons and generalizability across contemporary ACS cohorts.

### 3.4. Myeloperoxidase and Neutrophil Activation

Myeloperoxidase (MPO) represents another promising marker of residual inflammatory risk. MPO is a haemoprotein released predominantly by activated neutrophils and monocytes and plays a central role in oxidative stress and vascular inflammation. Through generation of reactive oxidant species, MPO promotes oxidation of LDL particles, endothelial dysfunction, degradation of nitric oxide, and destabilization of atherosclerotic plaques. These effects position MPO at the intersection of inflammation, oxidative stress, and thrombosis. Several studies have demonstrated that elevated MPO concentrations are independently associated with increased mortality and adverse cardiovascular outcomes following ACS. Importantly, these associations persist after adjustment for both troponin and hsCRP, suggesting that MPO captures distinct biological information not reflected by markers of myocardial injury or systemic inflammation alone [51,52]. Because MPO reflects active leukocyte-mediated vascular injury, it may be particularly useful for identifying patients characterized by ongoing plaque instability and heightened residual inflammatory risk despite apparently successful treatment of the acute ischemic event.

The clinical utility of MPO is, however, constrained by the absence of standardized assay platforms and validated cutoffs for routine use in ACS.

### 3.5. Clonal Haematopoiesis of Indeterminate Potential (CHIP)

An emerging and biologically plausible modifier of residual inflammatory risk is represented by clonal haematopoiesis of indeterminate potential (CHIP), in which somatic mutations in haematopoietic stem cells, particularly those reaching a variant allele frequency ≥2%, have been associated with enhanced inflammatory signalling and, in preliminary studies, with adverse cardiovascular outcomes after ACS [53,54]; however, evidence remains heterogeneous and prospective validation in dedicated ACS cohorts is required.

### 3.6. Anti-Inflammatory Therapies

The recognition of inflammation as a major contributor to residual cardiovascular risk has stimulated considerable interest in anti-inflammatory therapeutic strategies. Several pharmacological approaches have been evaluated in ACS and chronic coronary disease with varying degrees of success. Colchicine has received particular attention because of its broad anti-inflammatory effects and favorable cost profile. The Colchicine Cardiovascular Outcomes Trial (COLCOT) trial demonstrated that low-dose colchicine initiated after myocardial infarction reduced recurrent cardiovascular events [55]. Similarly, the Low-Dose Colchicine (LoDoCo2) trial reported significant reductions in cardiovascular outcomes among patients with chronic coronary disease treated with colchicine [56]. However, enthusiasm has been tempered by more recent evidence. The CLEAR SYNERGY (OASIS-9) trial, the largest randomized evaluation of colchicine following myocardial infarction, failed to demonstrate reductions in major adverse cardiovascular events or recurrent ischemic outcomes [57]. These conflicting results highlight the complexity of inflammatory pathways and suggest that patient selection, timing of intervention, and specific inflammatory phenotypes may substantially influence treatment efficacy. The most compelling evidence supporting inflammation as a therapeutic target remains the CANTOS trial. In patients with prior myocardial infarction and persistent inflammatory activation, canakinumab significantly reduced recurrent cardiovascular events without altering lipid levels. The greatest benefits were observed for recurrent myocardial infarction and composite cardiovascular endpoints. However, these benefits were offset by increased risks of serious infections and sepsis, and no reduction in all-cause mortality was observed [43]. Taken together, available evidence suggests that inflammation is not merely a biomarker of risk but a biologically relevant therapeutic target. Nevertheless, the role of anti-inflammatory therapy in routine post-ACS management remains incompletely defined. At present, inflammatory biomarkers may be most useful for identifying patients with heightened residual risk who could potentially benefit from future personalized therapeutic approaches targeting specific inflammatory pathways. Overall, inflammation represents one of the most important mechanisms contributing to residual cardiovascular risk after ACS. Biomarkers such as hsCRP, IL-6, leukocyte-derived indices, and MPO provide complementary prognostic information beyond traditional risk factors and support the concept that effective risk stratification must extend beyond cholesterol and clinical characteristics alone.

## 4. Myocardial Injury and Adverse Ventricular Remodelling

Myocardial injury and subsequent ventricular remodelling represent major determinants of long-term prognosis following ACS, particularly among patients presenting with myocardial infarction. Although restoration of coronary blood flow through timely reperfusion remains the cornerstone of treatment, the extent of myocardial damage sustained during the acute ischemic event initiates a complex cascade of biological responses that ultimately influence ventricular structure, function, and clinical outcomes. Following cardiomyocyte necrosis, inflammatory activation, extracellular matrix degradation, fibroblast proliferation, scar formation, and neurohormonal stimulation collectively contribute to the remodelling process. While these mechanisms initially serve reparative functions, excessive or maladaptive remodelling may result in progressive ventricular dilatation, impaired systolic performance, arrhythmogenesis, and the development of heart failure, which remains one of the principal causes of long-term morbidity and mortality after ACS. Cardiac imaging modalities, particularly echocardiography and cardiac magnetic resonance (CMR), provide detailed assessment of infarct size, left ventricular function, microvascular obstruction, intramyocardial haemorrhage, and structural remodelling. However, imaging alone cannot fully capture the dynamic biological processes occurring during myocardial injury and repair. Consequently, considerable interest has focused on circulating biomarkers capable of reflecting different aspects of myocardial damage, ventricular stress, inflammation, fibrosis, and remodelling (Table 3).

### 4.1. Cardiac Troponins

Cardiac troponins remain the reference biomarkers for the detection of myocardial injury and are central to the diagnosis of myocardial infarction. High-sensitivity cardiac troponin assays have revolutionized ACS management by enabling earlier and more accurate detection of myocardial necrosis and are currently recommended by international guidelines as the preferred biomarkers for diagnosis and risk assessment [3,4]. Beyond their diagnostic role, cardiac troponins are among the most powerful prognostic biomarkers available in ACS. Numerous studies have demonstrated strong associations between increasing troponin concentrations and adverse clinical outcomes, including mortality, recurrent myocardial infarction, cardiogenic shock, cardiac arrest, and heart failure. Importantly, prognostic information is preserved across the entire spectrum of troponin concentrations, indicating that risk increases progressively rather than merely above diagnostic thresholds [58,59,60]. The biological explanation for this association is intuitive. Troponin release reflects the magnitude of cardiomyocyte injury, which influences infarct size, residual ventricular function, arrhythmogenic substrate formation, and susceptibility to adverse remodelling. Persistent elevation of troponin concentrations after apparently successful revascularization may further identify patients experiencing ongoing myocardial injury or incomplete recovery and has been associated with particularly unfavourable long-term outcomes [61]. Importantly, troponins represent not only prognostic markers but also predictive biomarkers of treatment benefit. Several studies have shown that patients with elevated troponin concentrations derive greater benefit from early invasive management strategies and more intensive antithrombotic therapy than troponin-negative individuals [62,63,64,65]. Consequently, troponin occupies a unique position in ACS management by simultaneously informing diagnosis, prognosis, and therapeutic decision-making. Nevertheless, troponin primarily reflects the extent of myocardial necrosis and provides limited information regarding the subsequent biological processes that determine ventricular remodelling and heart failure development. This limitation has stimulated interest in additional biomarkers that capture complementary aspects of post-infarction pathophysiology.

### 4.2. Natriuretic Peptides: BNP and Nt-proBNP

B-type natriuretic peptide (BNP) and its inactive cleavage fragment N-terminal pro-B-type natriuretic peptide (NT-proBNP) are released predominantly by ventricular cardiomyocytes in response to increased wall stress and myocardial stretch [66]. Although traditionally viewed as markers of hemodynamic overload and heart failure, myocardial ischemia itself may directly stimulate natriuretic peptide synthesis, explaining their frequent elevation during ACS [67]. Among biomarkers of myocardial stress and remodelling, NT-proBNP is arguably the most extensively validated prognostic marker after ACS [68,69,70,71]. Numerous studies have consistently demonstrated independent associations between elevated NT-proBNP concentrations and increased risks of cardiovascular mortality, all-cause mortality, heart failure, and recurrent ischemic events. Importantly, these associations persist after adjustment for traditional risk factors, troponin concentrations, inflammatory biomarkers, and indices of renal dysfunction [67]. The prognostic strength of NT-proBNP appears particularly pronounced for mortality and heart failure outcomes [68]. This likely reflects its ability to integrate information from several pathophysiological domains simultaneously, including infarct size, ventricular dysfunction, elevated filling pressures, neurohormonal activation, and progressive remodelling. Consequently, NT-proBNP provides a more comprehensive assessment of post-infarction myocardial stress than markers of necrosis alone. Importantly, contemporary studies have demonstrated that NT-proBNP adds incremental prognostic information beyond established clinical risk scores such as GRACE and C-ACS, with enhanced net reclassification improvement (NRI) and integrated discrimination improvement suggesting potential utility in refining current risk prediction models [69,72]. Elevated concentrations have also been associated with a higher incidence of post-ACS complications including acute heart failure, recurrent myocardial infarction, ventricular aneurysm formation, and arrhythmias [71]. These observations support the concept that NT-proBNP serves as an integrated biomarker of ventricular vulnerability, identifying patients at increased risk of progressive myocardial dysfunction despite successful treatment of the acute ischemic event.

Nonetheless, NT-proBNP concentrations are substantially influenced by age, sex, atrial fibrillation, renal dysfunction, obesity, acute haemodynamic status, and the timing of sampling relative to reperfusion and initiation of heart failure therapy [73,74]; these factors may affect absolute concentrations and must be considered when interpreting prognostic associations.

### 4.3. Soluble Suppression of Tumorigenicity-2 (sST2)

Among emerging biomarkers of myocardial remodelling, soluble suppression of tumorigenicity-2 (sST2) has attracted considerable attention. sST2 belongs to the interleukin-1 receptor family and exists in both transmembrane and soluble forms. The membrane-bound receptor (ST2L) interacts with interleukin-33 (IL-33) to exert cardioprotective effects including attenuation of inflammation, fibrosis, hypertrophy, and adverse remodelling [75]. In contrast, circulating sST2 functions as a decoy receptor that binds IL-33 and prevents activation of these protective signalling pathways [75,76,77,78]. It should be noted, however, that sST2 functions in this review primarily as a prognostic biomarker reflecting the activation of this pathway under conditions of myocardial stress and injury, rather than as a demonstrated causal mediator of adverse outcomes in humans. In fact, elevated sST2 concentrations reflect disruption of endogenous mechanisms limiting myocardial injury and fibrosis. Initially developed as a biomarker in heart failure, sST2 has subsequently demonstrated substantial prognostic value in ACS populations. Multiple meta-analyses have consistently shown that elevated sST2 concentrations are independently associated with increased risks of all-cause mortality, cardiovascular mortality, heart failure, and major adverse cardiovascular events following ACS [79,80,81]. Interestingly, the prognostic relationship appears strongest for mortality and heart failure outcomes rather than recurrent myocardial infarction [82]. This pattern supports the hypothesis that sST2 primarily reflects myocardial injury, inflammation, fibrosis, and adverse ventricular remodelling rather than residual ischemic risk. Furthermore, evidence suggests that the prognostic value of sST2 is particularly evident in ACS populations and less pronounced in stable coronary artery disease, highlighting its relevance in acute cardiovascular injury [80]. Several studies have also demonstrated associations between elevated sST2 concentrations and subsequent left ventricular remodelling [82]. This finding is especially intriguing because it suggests that sST2 may identify maladaptive biological processes before structural changes become clinically apparent. Consequently, sST2 has emerged as one of the most promising biomarkers for detecting patients at risk of developing heart failure following ACS.

The prognostic value of sST2, however, should be interpreted in the context of its lack of a universally validated cut-off in ACS populations and the influence of acute inflammatory stimuli on its circulating concentrations, which may reflect systemic inflammation rather than myocardial remodelling specifically.

Formal assessment of incremental discriminative performance has nevertheless been demonstrated in a large prospective CAD cohort, inclusive of ACS patients, in which the addition of sST2 to established clinical risk factors significantly improved the C-index for all-cause mortality with significant a net reclassification improvement and integrated discrimination improvement, supporting its incremental prognostic value beyond conventional risk assessment, though replication in dedicated contemporary ACS cohorts remains warranted [83].

### 4.4. Growth Differentiation Factor-15

Growth differentiation factor-15 (GDF-15) is a stress-responsive cytokine belonging to the transforming growth factor-β superfamily. Under physiological conditions, circulating concentrations are low, but expression increases markedly in response to ischemia, oxidative stress, inflammation, mechanical stretch, and neurohormonal activation [84]. As a result, GDF-15 integrates signals from multiple pathophysiological pathways involved in cardiovascular disease. Unlike many biomarkers that primarily reflect a single biological process, GDF-15 appears to function as a broad indicator of cellular stress and tissue injury. This characteristic may explain its particularly strong and consistent prognostic performance across diverse cardiovascular populations. Substantial evidence supports the prognostic value of GDF-15 after ACS. Elevated concentrations have been associated with increased risks of cardiovascular mortality, all-cause mortality, recurrent myocardial infarction, stroke, and major bleeding events, independently of traditional clinical variables and established biomarkers [85,86]. Notably, few biomarkers simultaneously predict both ischemic and bleeding complications, making GDF-15 especially attractive in a clinical setting where balancing ischemic protection against bleeding risk remains a central challenge. The prognostic utility of GDF-15 extends beyond isolated risk prediction. Studies have demonstrated that incorporation of GDF-15 into multimarker strategies improves risk discrimination beyond traditional models and established biomarkers such as troponin and NT-proBNP. Indeed, GDF-15 has been successfully integrated into several biomarker-based risk prediction models for ACS [5,87]. Importantly, formal assessments of incremental discriminative value have confirmed that GDF-15 adds meaningful prognostic information beyond established risk tools: in a prospective ACS cohort, GDF-15 outperformed GRACE 2.0 for three-year mortality prediction (AUC 0.82 vs. 0.76; *p* = 0.001) and its addition to GRACE 2.0 further improved discrimination of all-cause death [88]. In addition to recurrent ischemic events, elevated GDF-15 has been associated with adverse ventricular remodelling and heart failure development [89,90]. These observations likely reflect the biomarker’s ability to capture multiple processes simultaneously, including inflammation, oxidative stress, tissue injury, and neurohormonal activation. Although the precise biological role of GDF-15 remains incompletely understood, current evidence strongly supports its value as an integrative marker of residual cardiovascular risk. It is important to note that whether GDF-15 directly contributes to adverse remodelling and post-ACS outcomes or, more likely, serves primarily as an integrated readout of the cumulative burden of ischemic injury, neurohormonal activation, and systemic inflammation, remains to be established. Nevertheless, its consistent association with adverse outcomes positions it among the most promising emerging biomarkers in contemporary ACS risk stratification.

### 4.5. A Multimarker Perspective on Myocardial Injury and Remodelling

The biomarkers discussed above reflect complementary aspects of post-infarction biology. Troponin quantifies the extent of cardiomyocyte necrosis, NT-proBNP captures ventricular stress and neurohormonal activation, sST2 reflects inflammatory and fibrotic remodelling processes, and GDF-15 integrates information regarding cellular stress, inflammation, and tissue injury. Rather than competing biomarkers, these markers should be viewed as components of a multidimensional framework describing different stages of myocardial injury and recovery. Their combined use may provide a more comprehensive assessment of risk than any single biomarker alone. Indeed, emerging evidence suggests that multimarker approaches incorporating markers of necrosis, inflammation, ventricular stress, and fibrosis may substantially improve identification of patients at risk of adverse remodelling, heart failure, and cardiovascular death following ACS. As the field moves toward increasingly personalized cardiovascular care, integration of these biomarkers with advanced imaging techniques and clinical risk scores may represent an important step toward more precise risk stratification and individualized post-ACS management.

## 5. Renal Dysfunction and the Cardiorenal Axis

Renal dysfunction is among the most powerful and consistently validated predictors of adverse outcomes following ACS. Both pre-existing chronic kidney disease (CKD) and acute kidney injury (AKI) occurring during hospitalization are associated with substantially increased risks of mortality, recurrent ischemic events, heart failure, and bleeding complications. Despite major advances in contemporary ACS management, the prognostic burden associated with impaired renal function remains remarkably persistent. The importance of renal dysfunction extends beyond its role as a comorbidity. Increasing evidence suggests that kidney impairment participates directly in the pathophysiological processes underlying adverse cardiovascular outcomes through complex interactions involving neurohormonal activation, endothelial dysfunction, inflammation, oxidative stress, fibrosis, and thrombosis. Consequently, renal biomarkers provide prognostic information that reflects not only kidney function itself but also broader systemic processes contributing to residual cardiovascular risk (Table 4).

### 5.1. Prognostic Impact of Renal Dysfunction in ACS

Chronic kidney disease is highly prevalent among patients presenting with ACS and has consistently emerged as an independent prognostic factor for both short- and long-term adverse outcomes. Large registries and prospective studies have demonstrated that reduced estimated glomerular filtration rate (eGFR) is associated with increased risks of in-hospital mortality, cardiovascular death, recurrent myocardial infarction, and heart failure across the entire spectrum of ACS presentations. Importantly, the relationship between renal function and prognosis appears continuous rather than threshold dependent [91,92,93,94]. Even modest reductions in eGFR have been associated with progressively increasing cardiovascular risk, suggesting that renal dysfunction represents a graded determinant of prognosis rather than simply a marker of advanced disease. Beyond pre-existing CKD, AKI represents a frequent complication of ACS and carries substantial prognostic implications. The development of AKI during hospitalization has been associated with increased mortality both during the index admission and throughout long-term follow-up [95]. The prognostic impact appears particularly pronounced among patients experiencing moderate or severe AKI, although uncertainty remains regarding the significance of milder forms of renal injury [96]. The relationship between cardiac and renal dysfunction is best understood through the framework of cardiorenal syndromes. In ACS, acute cardiac dysfunction may precipitate renal injury through reductions in cardiac output, renal hypoperfusion, venous congestion, and neurohormonal activation [97]. Conversely, renal dysfunction may further worsen cardiovascular status by promoting sodium retention, volume overload, endothelial dysfunction, inflammation, oxidative stress, and maladaptive neurohormonal responses. Several mechanisms amplify this interaction in ACS. Reduced renal perfusion secondary to myocardial dysfunction, activation of the renin–angiotensin–aldosterone system (RAAS), sympathetic nervous system stimulation, systemic inflammatory activation, and exposure to iodinated contrast during coronary angiography all contribute to renal injury. In patients with pre-existing CKD, chronic accumulation of uremic toxins further accelerates vascular dysfunction and promotes a complex pro-inflammatory and prothrombotic state [97,98,99]. One of the most clinically relevant consequences of renal dysfunction is its contribution to the treatment-risk paradox. Despite experiencing substantially higher event rates, patients with impaired renal function are less likely to undergo invasive management and frequently receive less intensive secondary prevention therapy [92]. Concerns regarding bleeding complications, contrast-induced nephropathy, altered drug pharmacokinetics, and procedural risk often lead clinicians to adopt more conservative approaches. Ironically, this therapeutic de-escalation frequently occurs in the very patients who have the greatest absolute potential to benefit from aggressive risk reduction strategies.

### 5.2. Serum Creatinine and Estimated Glomerular Filtration Rate

Serum creatinine and creatinine-derived estimates of glomerular filtration rate remain the foundation of renal assessment in clinical practice. Their prognostic significance in ACS has been demonstrated repeatedly across multiple populations and clinical settings, making them among the most extensively validated renal biomarkers available. Reduced eGFR consistently predicts mortality, recurrent cardiovascular events, and heart failure following ACS, even after adjustment for traditional cardiovascular risk factors [91,92]. Furthermore, recent evidence suggests that dynamic changes in renal function following myocardial infarction may provide prognostic information beyond baseline measurements alone. Patients exhibiting progressive deterioration in kidney function after ACS appear to experience particularly unfavourable long-term outcomes [100]. Despite their widespread use, creatinine-based estimates possess important limitations. Serum creatinine is influenced by numerous non-renal factors including muscle mass, age, sex, nutritional status, and physical activity. Consequently, creatinine-based eGFR may underestimate risk in frail or sarcopenic individuals, who often represent a particularly vulnerable subgroup within the ACS population. These limitations have stimulated interest in alternative renal biomarkers capable of providing more accurate assessment of kidney function and more refined cardiovascular risk stratification.

### 5.3. Cystatin C: An Emerging and Potentially Superior Biomarker

Among emerging renal biomarkers, cystatin C has attracted considerable attention as a potentially superior marker of kidney function and cardiovascular risk. Cystatin C is a low-molecular-weight cysteine protease inhibitor produced at a relatively constant rate by all nucleated cells and freely filtered by the glomerulus without significant tubular secretion. Unlike creatinine, circulating cystatin C concentrations are substantially less influenced by muscle mass, nutritional status, age, or sex. As a result, cystatin C may provide a more accurate estimate of glomerular filtration, particularly in elderly, frail, malnourished, or sarcopenic patients, populations commonly encountered after ACS. Evidence supporting the prognostic superiority of cystatin C has emerged from large population studies demonstrating improved risk stratification when kidney function is assessed using cystatin C rather than creatinine alone. Individuals reclassified into lower eGFR categories based on cystatin C measurements exhibit substantially higher risks of mortality and cardiovascular events than suggested by creatinine-derived estimates [101]. Importantly, the prognostic value of cystatin C has also been specifically confirmed in ACS populations. Recent meta-analyses have demonstrated independent associations between elevated cystatin C concentrations and increased risks of major adverse cardiovascular events, heart failure, stroke, and mortality following ACS [102,103]. These associations persist even after adjustment for conventional measures of renal function, suggesting that cystatin C captures clinically relevant biological information not fully reflected by creatinine. The mechanisms underlying these associations may extend beyond renal function alone. proposed pathways include direct modulation of vascular matrix degradation, promotion of atherogenic inflammation, and amplification of metabolic dysregulation raising the possibility that the biomarker reflects broader pathophysiological processes contributing to cardiovascular risk. Despite encouraging evidence, important questions remain unanswered. It should be noted that, despite its advantages over creatinine, cystatin C is not a purely renal marker: its concentrations are influenced by inflammation, adiposity, smoking, thyroid dysfunction, glucocorticoid exposure. Moreover, no universally accepted cystatin C threshold exists for risk stratification in contemporary ACS populations, and the optimal timing of measurement remains uncertain. Furthermore, whether cystatin C-guided management strategies can improve clinical outcomes has yet to be demonstrated. Nevertheless, current evidence positions cystatin C among the most promising renal biomarkers for future integration into multidimensional ACS risk assessment models.

### 5.4. Albuminuria and Systemic Vascular Injury

Albuminuria has long been recognized as a marker of kidney damage, but increasing evidence suggests that it may also serve as an indicator of diffuse endothelial dysfunction and systemic vascular injury. Through disruption of the glomerular filtration barrier, albuminuria reflects widespread microvascular damage extending beyond the kidney itself. Several studies have investigated its prognostic significance in ACS. Elevated urinary albumin excretion has been associated with increased long-term mortality following acute coronary events [104] supporting the hypothesis that albuminuria identifies patients with extensive vascular pathology and heightened residual cardiovascular risk However, evidence regarding its independent contribution to cardiovascular risk prediction remains somewhat conflicting. While some investigations have demonstrated strong associations with adverse outcomes, other analyses suggest that much of its prognostic value may be attributable to its close relationship with traditional cardiovascular risk factors such as hypertension, diabetes, and CKD [105]. Consequently, whether albuminuria provides substantial incremental prognostic information beyond these established factors remains uncertain.

### 5.5. Neutrophil Gelatinase-Associated Lipocalin

Neutrophil gelatinase-associated lipocalin (NGAL) has emerged as a promising biomarker of acute tubular injury. Released by injured renal tubular epithelial cells and activated neutrophils, NGAL rises rapidly following kidney injury, often preceding increases in serum creatinine by 24 to 48 h This early response has generated considerable interest in ACS, where timely identification of AKI may facilitate preventive interventions and optimize management. In addition to predicting contrast-associated kidney injury following PCI [106], elevated NGAL concentrations have been associated with increased long-term cardiovascular risk. Studies have demonstrated independent associations between higher baseline NGAL levels and major adverse cardiovascular events after ACS [107]. These findings suggest that NGAL may provide information extending beyond renal injury alone, potentially reflecting inflammatory activation and systemic vulnerability. Although NGAL remains primarily a research biomarker, its ability to detect subclinical renal injury before conventional markers become abnormal highlights its potential value in future cardiorenal risk assessment strategies.

### 5.6. The Cardiorenal Axis as a Multidimensional Risk Domain

The growing body of evidence linking renal dysfunction to adverse cardiovascular outcomes supports the concept that kidney injury should be viewed not merely as a comorbidity but as a central component of residual risk after ACS. Traditional markers such as creatinine and eGFR remain indispensable, emerging biomarkers including cystatin C and NGAL may provide additional prognostic insights reflecting the mechanistic pathways linking renal dysfunction to adverse cardiovascular outcomes, without necessarily implying direct causal participation in those mechanisms. Importantly, the cardiorenal axis intersects with many of the other risk domains discussed in this review, including inflammation, neurohormonal activation, myocardial remodelling, and metabolic dysfunction. Consequently, renal biomarkers often function as integrative indicators of global biological vulnerability rather than isolated measures of kidney function. As precision medicine approaches continue to evolve, incorporation of both traditional and emerging renal biomarkers into multimarker risk stratification models may improve identification of patients at highest risk of recurrent events and help guide more individualized secondary prevention strategies following ACS.

## 6. Obesity and Visceral Adiposity

Obesity is a major contributor to cardiovascular disease [108] and is associated with multiple mechanisms that promote atherosclerosis, including insulin resistance, dyslipidaemia, chronic low-grade inflammation, endothelial dysfunction, oxidative stress, and adverse metabolic remodelling. Excess adipose tissue contributes to the development and progression of coronary artery disease through complex endocrine and inflammatory pathways that influence both vascular biology and myocardial function. Despite these well-established associations, the relationship between obesity and prognosis after ACS remains surprisingly complex. While obesity increases the risk of developing cardiovascular disease, numerous studies have reported paradoxically better outcomes among overweight and obese patients following ACS [109]. This phenomenon, commonly referred to as the “obesity paradox,” remains one of the most debated observations in cardiovascular medicine. Importantly, increasing evidence suggests that this apparent paradox may largely reflect the limitations of body mass index (BMI) as a measure of adiposity [108]. BMI provides a simple estimate of body weight relative to height but fails to distinguish between fat and lean mass, does not account for body fat distribution, and provides limited information regarding the metabolic activity of adipose tissue. Consequently, individuals with identical BMI values may exhibit profoundly different cardiovascular risk profiles. These limitations have stimulated growing interest in markers of visceral adiposity, which may provide more biologically relevant information regarding residual cardiovascular risk after ACS.

### 6.1. The Obesity Paradox

Several observational studies have reported lower mortality rates among overweight and moderately obese patients following myocardial infarction and ACS compared with individuals classified as having normal body weight [109]. Although the underlying mechanisms remain incompletely understood, several explanations have been proposed. One possibility is that obese patients tend to present at a younger age and often have fewer age-related comorbidities than leaner patients experiencing ACS. Earlier presentation may result in greater physiological reserve and improved tolerance of acute cardiovascular injury. Furthermore, lower BMI categories frequently include frail individuals, patients with chronic illness, sarcopenia, cachexia, or occult disease states that independently worsen prognosis. Even after adjustment for several confounding factors, however, some studies have continued to demonstrate a protective association between higher BMI and post-ACS outcomes [109]. This observation has led investigators to question whether BMI accurately captures the aspects of adiposity most relevant to cardiovascular risk. Increasing evidence suggests that body fat distribution may be substantially more important than overall body weight. In particular, accumulation of visceral adipose tissue appears to be more strongly associated with metabolic dysfunction, inflammation, and adverse cardiovascular outcomes than total adiposity alone.

### 6.2. Waist Circumference and Abdominal Obesity

Among measures of visceral adiposity, waist circumference has emerged as a particularly informative marker of cardiovascular risk. Unlike BMI, waist circumference reflects central fat accumulation and provides a surrogate measure of metabolically active abdominal adiposity. Abdominal obesity is associated with insulin resistance, systemic inflammation, endothelial dysfunction, adverse lipid profiles, and increased secretion of pro-inflammatory adipokines. Through these mechanisms, visceral fat contributes to accelerated atherosclerosis and persistent residual cardiovascular risk even among patients receiving optimal medical therapy.

Evidence supporting the prognostic value of abdominal obesity has emerged from large post-myocardial infarction cohorts. In the SWEDEHEART registry, increased waist circumference was independently associated with recurrent atherosclerotic cardiovascular events following myocardial infarction, even after adjustment for BMI and traditional cardiovascular risk factors [110]. These findings suggest that abdominal obesity captures clinically relevant information not reflected by BMI alone and may therefore represent a superior marker of residual cardiovascular risk.

Importantly, these observations provide a potential explanation for the obesity paradox. While BMI may inadequately distinguish between metabolically healthy and metabolically unhealthy obesity, measures of central adiposity appear more closely linked to the biological pathways driving recurrent cardiovascular events.

### 6.3. Epicardial Adipose Tissue

Among emerging markers of visceral adiposity, epicardial adipose tissue (EAT) has generated particular interest. EAT is a metabolically active visceral fat depot located between the myocardium and visceral pericardium, in direct anatomical contact with the coronary arteries and underlying myocardium.

Unlike subcutaneous adipose tissue, EAT exhibits unique biological properties. It functions as an active endocrine and paracrine organ capable of secreting a broad range of cytokines, adipokines, chemokines, and inflammatory mediators. Through these pathways, EAT may directly influence coronary atherosclerosis, plaque vulnerability, myocardial injury, and ventricular remodelling [111]. Experimental and clinical evidence increasingly supports a role for EAT in cardiovascular disease progression. Increased EAT thickness and volume have been associated with greater coronary atherosclerotic burden, more extensive coronary artery disease, and higher prevalence of vulnerable plaque characteristics [112]. These observations suggest that EAT may serve as both a marker and mediator of adverse cardiovascular biology. Particularly intriguing is the potential relationship between EAT and ACS outcomes. Observational studies have demonstrated associations between increased EAT and adverse reperfusion phenomena, including the no-reflow phenomenon in STEMI patients. Furthermore, greater EAT burden has been linked to increased mortality following AMI [113]. Several mechanisms may explain these associations. Through secretion of inflammatory cytokines and profibrotic mediators, EAT may amplify local coronary inflammation and promote plaque instability. In addition, EAT-derived inflammatory signals may contribute to myocardial injury, microvascular dysfunction, and adverse ventricular remodelling following infarction.

Nevertheless, current evidence remains largely observational. Although the biological rationale supporting EAT as a prognostic biomarker is compelling, available studies remain relatively small and heterogeneous. Moreover, a critical limitation is the methodological heterogeneity of EAT measurement: echocardiographic thickness, CT-derived volume, CT attenuation, and CMR-derived mass quantify different aspects of adipose tissue, are not interchangeable, and differ substantially in reproducibility, acquisition protocols, anatomical landmarks, and threshold definitions. Consequently, whether EAT provides clinically meaningful prognostic information beyond established risk markers remains uncertain.

### 6.4. Visceral Adiposity as an Emerging Risk Domain

The evolving understanding of obesity after ACS highlights an important shift in cardiovascular risk assessment. Rather than focusing exclusively on overall body weight, increasing attention is being directed toward the location, composition, and biological activity of adipose tissue. Measures of visceral adiposity, particularly waist circumference and epicardial adipose tissue, appear to provide more relevant information regarding residual cardiovascular risk than BMI alone. These markers integrate several pathophysiological pathways discussed throughout this review, including inflammation, insulin resistance, endothelial dysfunction, and myocardial remodelling. Although further research is required before visceral adiposity markers can be routinely incorporated into clinical risk models, current evidence suggests that they represent promising additions to multidimensional risk stratification strategies. Future studies will determine whether targeting visceral adiposity itself can modify cardiovascular outcomes and whether imaging-based assessment of adipose tissue can improve identification of high-risk patients following ACS.

## 7. Platelet-Derived Prognostic Markers

Platelet activation has a central role in the pathogenesis of ACS, contributing to thrombus formation and propagation, through its engagement in coagulation cascade, and favouring adverse post-infarction remodelling through the release of pro-inflammatory mediators. Among platelet-derived indices, the platelet-to-lymphocyte ratio (PLR), already discussed in the context of leukocyte-derived inflammatory markers, integrates platelet-mediated thrombotic burden with lymphocyte-mediated immune regulation, and its independent prognostic value after ACS has been confirmed in recent meta-analytic evidence [49,113,114]. Mean platelet volume (MPV), a surrogate of platelet size and prothrombotic potential, has shown consistent prognostic value for short-term mortality and long-term MACE across CAD and ACS populations, although results for long-term mortality remain contrasting between meta-analyses [115,116].

Tissue factor (TF), the primary initiator of the extrinsic coagulation cascade, has been identified in two distinct compartments with potentially different prognostic significance: a soluble form carried by circulating microparticles, and a platelet-associated membrane form reflecting direct platelet-coagulation crosstalk. While the circulating form showed mixed results as to its association to the poor prognosis in ACS [117,118], in the prospective TRIT-ONE study [119] enrolling 527 CAD patients, including 149 with ACS, platelet-associated TF expression was the only biomarker to independently predict both all-cause mortality and cardiovascular mortality at 5-year follow-up.

However, these findings should be interpreted with caution. Current evidence is largely derived from a single prospective CAD cohort in which only a subset of patients presented with ACS, and independent validation in dedicated ACS populations is still lacking. Moreover, the assessment of platelet-associated TF relies on flow cytometry-based methodologies that remain technically demanding and have not yet been standardized for routine clinical application.

Collectively, platelet-derived biomarkers represent a promising domain of prognostic risk stratification after ACS. Although these observations support the biological relevance of persistent platelet activation after ACS, there is currently no evidence from biomarker-guided randomized trials demonstrating that platelet-derived microparticles or platelet-associated TF can identify patients who benefit from intensified or prolonged antithrombotic therapy. Their potential role in guiding treatment therefore remains hypothetical and requires prospective validation.

Consequently, platelet-derived microparticles and platelet-associated TF should currently be regarded as promising investigational prognostic biomarkers rather than clinically actionable biomarkers for therapeutic decision-making.

## 8. Vitamin D Deficiency

Even if not usually included among traditional cardiovascular risk factors, a large body of evidence has emerged linking vitamin D deficiency to cardiovascular disease in recent decades.

Experimental and preclinical models suggest that vitamin D exerts pleiotropic cardiovascular effects mediated through the vitamin D receptor (VDR) and the enzyme 1α-hydroxylase, both expressed by cardiomyocytes, vascular smooth muscle cells, fibroblasts, and endothelial cells. In these models, VDR activation has been shown to suppress RAAS signalling, inhibit angiotensin II generation, and modulate metalloproteinase activity, mechanisms that may collectively attenuate myocardial hypertrophy, fibrosis, and adverse remodelling. Whether these effects translate to clinically meaningful cardiovascular protection in humans, however, remains uncertain, as discussed below. Vitamin D deficiency has itself been independently associated with systemic hypertension, dyslipidaemia, and type 2 diabetes mellitus, further amplifying cardiovascular risk through indirect pathways [120,121,122,123,124].

Several observational studies have established an association between low vitamin D levels and adverse outcomes in ACS and AMI. Vitamin D levels were found to be significantly lower in patients with stable angina or AMI compared to controls, with a graded inverse relationship between vitamin D quartiles and AMI risk even after adjustment for major cardiovascular risk factors in large prospective cohorts [120]. In a prospective cohort of patients with ACS, severe vitamin D deficiency at admission was independently associated with a markedly higher risk of in-hospital cardiovascular mortality after full adjustment for clinical risk scores [125]. In a large AMI cohort followed for one year, low vitamin D levels emerged as a strong predictor of both all-cause mortality and rehospitalisation for acute heart failure, confirming its prognostic relevance beyond the acute event [126]. Similarly, in a large cohort of CAD patients undergoing PCI, vitamin D deficiency was independently associated with significantly higher rates of long-term all-cause mortality and MACE over multi-year follow-up [127].

Despite the strong biological rationale and consistent observational evidence, findings from randomized intervention trials remain inconclusive. Meta-analyses of randomized controlled trials have failed to demonstrate that vitamin D supplementation reduces major adverse cardiovascular events (MACE), myocardial infarction, or all-cause and cardiovascular mortality in the general population [128]. Among patients with established CAD, supplementation has shown modest favourable effects on diastolic blood pressure and parathyroid hormone levels but no significant impact on inflammatory biomarkers or lipid profiles [129]. In patients with ACS, a small randomized trial conducted in vitamin D-deficient individuals suggested that short-term vitamin D supplementation may attenuate adverse left ventricular remodelling, modulate profibrotic pathways, and potentially reduce the incidence of heart failure and MACE. However, these findings are limited by the modest sample size and require confirmation in adequately powered multicenter trials before any clinical implications can be drawn [130]. Overall, current evidence supports vitamin D deficiency as a prognostic marker reflecting the cumulative burden of cardiovascular risk, inflammation, and comorbidity, but does not support vitamin D supplementation as a strategy for cardiovascular risk reduction after ACS. The consistent failure of randomized trials to demonstrate benefit, despite robust observational associations, strongly suggests that low vitamin D levels function primarily as a marker of frailty and advanced disease rather than as a causal and modifiable determinant of adverse outcomes. Pending adequately powered trials enriched for vitamin D-deficient ACS populations, supplementation cannot currently be recommended for the purpose of improving cardiovascular prognosis.

## 9. Limitations of the Current Evidence

The evidence reviewed in this manuscript must be interpreted in the context of several important limitations. First, the studies supporting individual biomarkers are characterized by marked heterogeneity in study populations, spanning STEMI, NSTE-ACS, mixed ACS cohorts, patients with prior myocardial infarction, stable coronary artery disease populations, and patients undergoing elective or urgent PCI. In some instances, most notably the platelet-associated tissue factor data, ACS patients represent only a subset of the studied population, and findings from mixed CAD or chronic coronary disease cohorts should not be uncritically extrapolated to the contemporary ACS setting.

Second, outcome definitions across studies are substantially heterogeneous. The biomarkers reviewed here have been evaluated against all-cause mortality, cardiovascular mortality, recurrent myocardial infarction, heart failure, major bleeding, ventricular remodelling, no-reflow, acute kidney injury, and a variety of composite MACE endpoints, outcomes that represent fundamentally different pathophysiological processes and cannot be treated as interchangeable measures of prognostic value. Definitions of MACE differ across trials, follow-up durations range from in-hospital to over ten years, and biomarker sampling time points, whether at admission, at peak, at discharge, or during convalescence, carry different biological and prognostic meanings that are rarely systematically compared within individual studies.

Third, several biomarkers discussed in this review lack universally validated cut-offs in ACS cohorts, standardized assay platforms, and prospective evidence demonstrating that biomarker-guided management improves clinical outcomes. Turnaround time represents an additional constraint: biomarkers requiring specialized flow cytometry or high-complexity immunoassay platforms may not be available within the time-sensitive context of acute clinical decision-making. Cost considerations further limit adoption, particularly for markers whose incremental prognostic value over standard biochemical panels has not yet been formally demonstrated in cost-effectiveness analyses.

Fourth, formal assessments of incremental discriminative performance, including net reclassification improvement (NRI), integrated discrimination improvement (IDI), calibration statistics, and clinical decision curve analysis, are available for only a minority of the biomarkers. For most other biomarkers, however, ACS-specific contemporary cohorts are either absent or insufficient. Statistically significant independent associations, while biologically informative, do not in themselves justify clinical implementation; formal evidence of meaningful risk reclassification and favourable clinical decision curves, currently lacking for the majority of markers reviewed, represents a necessary prerequisite for translation into routine clinical practice.

## 10. Conclusions

Despite major advances in revascularization, antithrombotic therapy, and lipid lowering, patients with ACS continue to face substantial residual risks of recurrent ischemic events, heart failure, and cardiovascular death. The evidence reviewed highlights that prognosis after ACS is determined by multiple interconnected pathophysiological pathways, including lipid abnormalities, inflammation, myocardial injury and remodelling, renal dysfunction, and visceral adiposity. Several emerging biomarkers, including hsCRP, IL-6, NT-proBNP, sST2, GDF-15, and cystatin C, provide complementary prognostic information beyond traditional risk factors and established risk scores (Table 5). Among the emerging biomarkers discussed in this review, high-sensitivity troponin and NT-proBNP are currently incorporated into clinical guidelines and represent the only markers with established routine clinical utility for biomarker-based prognostic assessment in ACS. The remaining biomarkers discussed in this review should be regarded as promising adjunctive tools whose integration into clinical decision-making requires further prospective validation, including demonstration of incremental value over GRACE and TIMI in contemporary cohorts, which has already been explored for some biomarkers, particularly NT-proBNP and GDF-15, as well as assay standardization and evidence from biomarker-guided randomized trials.

Looking ahead, however, the individual biomarkers reviewed here are unlikely to fulfil their full prognostic potential in isolation. Increasing evidence suggests that multimarker panels, combining markers from complementary pathophysiological domains such as inflammation, myocardial stress, and renal dysfunction, outperform single-biomarker approaches in risk discrimination and reclassification. Integration with established clinical scores and advanced imaging parameters may further enhance predictive performance. High-dimensional omics approaches, including proteomic profiling, metabolomic signatures, transcriptomics, and integrated multi-omics strategies, are beginning to identify novel molecular signatures capable of refining post-ACS risk stratification, and are likely to define the next generation of precision medicine in this setting, though none have yet reached clinical validation.

Finally, artificial intelligence and machine learning algorithms capable of processing high-dimensional clinical, laboratory, imaging, and emerging omics data offer transformative potential for individualized risk stratification after ACS, as exemplified by machine learning frameworks already applied to imaging-based identification of patients at risk of rapid coronary atherosclerosis progression [131]. These approaches represent the most promising direction for future biomarker research and will ultimately require prospective validation in adequately powered, biomarker-enriched randomized trials before routine clinical implementation can be justified.

## Figures and Tables

**Table 1:** Traditional and Lipid-Related Prognostic Risk Factors After ACS.

Risk Factor	Pathophysiological Mechanism	Prognostic Association After ACS	Key Clinical Implication
LDL-C	Atherosclerosis progression, plaque instability, thrombosis	Higher LDL-C associated with increased recurrent ischemic risk; intensive LDL-C lowering reduces recurrent events	Primary therapeutic target; residual risk persists despite optimal control
Lipoprotein(a)	Pro-atherogenic, pro-inflammatory, pro-thrombotic effects	Associated with increased MACE and all-cause mortality	Emerging marker of residual lipid risk; therapeutic target under investigation
Smoking	Endothelial dysfunction, oxidative stress, platelet activation, inflammation	Increased recurrent MI and adverse ventricular remodelling	Smoking cessation remains a cornerstone of secondary prevention
Hypertension	Vascular remodelling, arterial stiffness, microvascular dysfunction	Increased long-term mortality and HF after MI	Marker of vascular vulnerability beyond infarct size
Diabetes Mellitus	Hyperglycemia, inflammation, microvascular dysfunction, diffuse CAD	Increased mortality across all ACS presentations; risk increases with disease duration	Represents a major determinant of residual cardiovascular risk

**Table 2:** Inflammatory Biomarkers, Residual Inflammatory Risk and Platelet activity markers.

Biomarker	Biological Pathway	Main Prognostic Associations	Potential Added Value/Clinical Considerations
hsCRP	Systemic inflammation; downstream IL-6 signaling	Mortality, recurrent ischemic events, HF, restenosis	Independent of LDL-C and troponin
IL-6	Upstream inflammatory signaling	Cardiovascular death, recurrent MI, HF	Reflects pathogenic inflammatory activity
Neutrophil-to-Lymphocyte Ratio (NLR)	Innate/adaptive immune imbalance	Mortality, reduced LVEF, remodelling, thrombus formation	Widely available and inexpensive
SIRI/SII	Integrated leukocyte activation	MACE prediction	May improve GRACE-based risk assessment
MPO	Oxidative stress and plaque destabilization	Mortality and recurrent ischemic events	Identifies plaque instability and residual inflammatory risk
Platelet-Associated Tissue Factor (TF)	Platelet-coagulation crosstalk; TF expression on activated platelet surface	All-cause and cardiovascular mortality at long-term follow-up in CAD including ACS	Only platelet marker independently predicting mortality in prospective cohort (TRIT-ONE); methodological standardization needed

**Table 3:** Biomarkers of Myocardial Injury and Adverse Ventricular Remodelling.

Biomarker	Principal Biological Signal	Prognostic Outcomes	Potential Clinical Role
High-Sensitivity Troponin	Myocardial necrosis	Mortality, recurrent MI, cardiogenic shock, HF	Diagnostic and prognostic biomarker
NT-proBNP	Ventricular stress and neurohormonal activation	Mortality, HF, recurrent ischemic events	Identifies patients at risk of HF development
sST2	Fibrosis, inflammation, adverse remodelling	Mortality, HF, MACE	Marker of ventricular remodelling
GDF-15	Cellular stress, oxidative injury, inflammation	Mortality, MI, stroke, major bleeding	Integrates ischemic and bleeding risk
Multimarker Approach	Combined injury, stress, fibrosis pathways	Improved risk discrimination	Future precision-medicine strategy

**Table 4:** Cardiorenal Dysfunction. Visceral Adiposity and endocrine domain in ACS.

Marker	Biological Domain	Prognostic Significance	Current Evidence
eGFR	Renal function	Strong predictor of mortality and MACE	Established prognostic marker
Acute Kidney Injury	Cardiorenal syndrome	Increased in-hospital and long-term mortality	Particularly relevant after PCI
Cystatin C	Renal dysfunction and systemic injury	MACE, HF, stroke, mortality	Potentially superior to creatinine
Albuminuria	Endothelial dysfunction	Long-term mortality	Incremental value remains debated
NGAL	Tubular injury	AKI prediction and MACE	Early marker of renal injury
Waist Circumference	Visceral adiposity	Recurrent ASCVD events after MI	Superior to BMI for residual risk
Epicardial Adipose Tissue	Local coronary inflammation	CAD burden, no-reflow, mortality	Promising but requires further validation
Vit. D Deficiency	Neuroendocrine/metabolic	Independently associated with mortality, HF rehospitalisation, and MACE after ACS	Observational evidence robust, interventional trial inconclusive

**Table 5:** Emerging Biomarkers Ranked by Clinical Readiness for ACS Risk Stratification.

Biomarker	Prognostic Association	Clinical Validation	Current Availability/Guideline Status	Clinical Implementation
Lp(a)	Strong	Extensive prospective evidence	Routinely measurable; guideline-supported for risk assessment	Risk refinement/secondary prevention
hsCRP	Strong	Extensive (large prospective cohorts; RCT post-hoc analyses)	Routinely available; not routinely recommended	Adjunctive risk stratification
IL-6	Moderate–Strong	Moderate (prospective cohorts; RCT substudies)	Available in specialized laboratories; not guideline-recommended	Research/adjunctive risk stratification
NLR/SIRI	Moderate–Strong	Moderate (multiple cohorts; meta-analyses)	Widely available as routine laboratory-derived indices; not guideline-recommended	Practical adjunct/research
MPO	Moderate	Limited (predominantly small cohorts)	Limited routine availability; not guideline-recommended	Research only
Platelet-associated TF	Preliminary	Limited (single prospective CAD cohort; ACS subset only)	Not routinely available; not guideline-recommended	Research only
Troponin (hs-cTn)	Strong	Extensive (large cohorts; registries; RCT substudies)	Routinely available; guideline-recommended	Established clinical use
NT-proBNP	Strong	Extensive (prospective cohorts; registries; RCT substudies)	Routinely available; guideline-supported for prognostic assessment	Established adjunctive prognostic use
sST2	Moderate–Strong	Moderate (prospective cohorts; meta-analyses)	Available but not routinely recommended	Adjunctive/research
GDF-15	Strong	Moderate (prospective cohorts; RCT substudies; meta-analyses)	Available in specialized laboratories; not guideline-recommended	Risk refinement/research
Creatinine/eGFR	Strong	Extensive (large registries; prospective cohorts; meta-analyses)	Routinely available; guideline-supported	Established clinical use
Cystatin C	Moderate–Strong	Moderate (prospective cohorts; meta-analyses)	Available in clinical laboratories; not routinely recommended for ACS risk stratification	Adjunctive/research
EAT	Moderate	Moderate (observational cohorts; imaging studies)	Imaging-dependent; not routinely recommended	Research/risk refinement
Vitamin D deficiency	Moderate	Limited (observational cohorts; small intervention studies)	Routinely measurable; not recommended for ACS prognostic stratification	Research/selected clinical assessment

## Data Availability

No new data were created or analyzed in this study. Data sharing is not applicable to this article.
